# “We Are at The Forefront of Rural Areas” Emergency Nurse's Experience During Pandemic: A Qualitative Study

**DOI:** 10.1089/heq.2021.0080

**Published:** 2021-12-06

**Authors:** Ronal Surya Aditya, Ah Yusuf, Daifallah M. Al Razeeni, Khaled Mohammed Al-Sayaghi, Fitriana Kurniasari Solikhah

**Affiliations:** ^1^ Sekolah Tinggi Ilmu Kesehatan (STIKes) Kepanjen Malang, Malang, Indonesia.; ^2^ Faculty of Nursing, Universitas Airlangga Surabaya, Surabaya, Indonesia.; ^3^ Department of EMS, Vice Dean for Academic Affair Prince Sultan Bin Abdalziz College for EMS (PSEMS), King Saud University (KSU), Riyadh, Saudi Arabia.; ^4^ College of Nursing, Taibah University, Madinah, Saudi Arabia.; ^5^ Poltekkes Kemenkes Malang, Malang, Indonesia.

**Keywords:** COVID-19, Emergency Public Health Center, nurse

## Abstract

**Purpose:** The pandemic makes everyone alert, including nurses who are in emergency rooms at community health centers, the importance of their experiences is a lesson for nurses to maintain themselves and be effectivein providing services. This study aimed to explore the experience of nurses who are in emergency rooms at rural area during the coronavirus disease pandemic.

**Methods:** This qualitative research was conducted based on the Nvivo 12 analysis method using in-depth semistructured interviews. Data saturation was reached after 20 interviews were completed. Data collection lasted for 1 month from February to March 2020.

**Results:** Semistructured interviews with 20 nurse participants obtained the following participant characteristics. Eight participants were males and 12 were females with an age ranged from 28 to 43 years (average age 36.4 years). The majority had vocational education (75%), with long experience that ranged from 5 to 15 years (average 11 years). The findings of four themes and seven subthemes. The theme of the findings is Expressions of care, Compliance increases using Personal Protective Equipment (PPE), Focus to refer to, Triage at the forefront.

**Conclusion:** This research reveals that Expressions of care, Compliance increases using PPE, Focus to refer to, Triage at the forefront is the main theme identified in this study. Further investigation of the readiness of nurses in handling patients in the emergency room is considered to be of benefit to the results of this study.

## Introduction

Coronavirus disease (COVID-19) is a respiratory infection caused by a new type of corona virus called Severe Acute Respiratory Syndrome Corona Virus 2 (SARS-CoV-2), previously known as “2019 novel coronavirus.”^1,2^ Chinese authorities isolated and identified COVID-19 virus on January 7, 2020 in Wuhan City, Hubei Province, China. COVID-19 virus can be transmitted through respiratory droplets and contact routes.^3,4^ The transmission can occur by direct contact with infected person and indirect contact with surfaces in the environment or with objects used on the infected person (e.g., thermometer or stethoscope) than touching eyes, nose, or mouth.^5,6^

As of October 6, 2021, COVID-19 cases in Indonesia were recorded at 4,223,094 people. Of that 4,052,300 people have recovered from COVID-19, 28,381 people are still undergoing treatment in hospitals or in self-isolation, whereas 142,413 others have died. As of October 6, 2021, COVID-19 cases in Indonesia were recorded at 4,223,094 people. Of that 4,052,300 people have recovered from COVID-19, 28,381 people are still undergoing treatment in hospitals or in self-isolation, whereas 142,413 others have died. The availability of isolation rooms in rural areas will be a solution to anticipate full hospitals due to the booming number of COVID-19 patients.^7^

A transmission of COVID-19 is more likely to occur at the emergency departments in the health care service. The emergency department is the first entrance to the patient, this makes the transmission between new undiagnosed patients and health care workers on duty very high. Among the emergency department health care workers who are vulnerable to COVID-19 transmission, nurses are the most vulnerable group.^8,9^ The risk of COVID-19 transmission to nurses in the emergency department is very high.^10^

Previous findings state that the transmission of COVID-19 to health care workers is caused by some factors such as they have inadequate protection, long-term exposure to patients who are directly infected, work pressure, and nonavailability of personal protective equipment (PPE).^11–14^ However, despite the application of complete personal protective equipment, still there are many health care workers who are vulnerable to contracting COVID-19. The cause of transmission of COVID-19 to health care workers is not only the transmission factor but also because of the emergency management system implemented during the pandemic. An appropriate emergency management system is needed for the health service system during the current pandemic.^9,14–16^

The geographical breadth of rural and remote Indonesia areas provide challenges in the provision of emergency care. People in rural and remote Indonesia are more likely to engage in behaviors associated with poorer health outcomes.^17^ In many of the smaller hospitals in rural and nonexistent remote areas: no medical personnel and patients are transferred to other facilities or medical personnel are called upon when patients are admitted to hospital or while traveling. In both cases, there is often a delay before medics arrive at the hospital to treat critically ill or injured patients.^18^ In smaller rural and remote hospitals, nurses are the majority of staff and on-site medical personnel are rare. In many cases, registered nurses are required to provide First-Line Emergency Care management in the absence of medical staff.^19^

There were limited studies that have examined experience of nurses who are in emergency rooms at rural area experience during the current pandemic in Indonesia; this study was carried out to explore the experience of emergency nurses during the COVID-19 pandemic. This research is expected to be useful for registered nurse who works in emergency departments.

## Methods

### Study design

The study adopted a qualitative explorative descriptive design using semistructured interviews with emergency nurses. The qualitative approach is suitable for exploring and understanding the emergency nurses' experiences during the COVID-19 pandemic. Qualitative approach provides maximal explanation in a way that cannot be obtained by quantitative approach.^20^ The Consolidated Criteria for Qualitative Research Reporting (COREQ) were used for reporting and the thoroughness and validity of the analysis.^21^

### Setting, sampling, and participant

A purposive sampling design was implemented for this study. Purposive sampling is used largely in exploratory research or field research, and unique cases are selected based on judgment for the unique qualities that these cases offer in addressing a research problem.^22^ Purposive sampling technique was used to recruit information-rich nurses from different public health centers in Malang district, East Java, Indonesia. Nurses with long experience in nursing (i.e., ≥1 year), who actually receive patients and provide care for patients in the emergency rooms during the COVID-19 pandemic, and willingness to participate in this study were eligible for participation in the study.

### Data collection

All researchers conducted interviews through online (zoom or Skype) were equally divided and experienced in conducting qualitative research. qualifications of master researchers, doctorates and professors. consists of four men and one woman, They conduct research acting as lecturers, researchers, and participants do not have family or other relationships, and participants know the purpose of the study. The researcher has no interest in this research. After making contact with the participants and introducing the researcher to them, the details of the interview method were explained to them.

Participation was anonymous and participants are assured that all personal information and interviews will be kept confidential. Data collection took place for 1 month from February to March 2020. The semistructured interviews were done on an individual bases through zoom due to the lock conditions. All interviews were recorded by digital voice recorders. The semistructured interview was guided by open-ended questions that allowed participant and interviewer to follow any interesting or relevant experience of enquiry.

The researcher began the interview by asking one general question about their experiences in the emergency room during the pandemic. Based on the results of the interview data, the probing questions for the co-construction of the next interview were revised. Immediately after each interview, the interview audio files were transcribed verbatim to maintain data integrity and reduce researcher bias. The interview duration varies between 30 and 65 min (average 47 min) depending on the conditions and interests, experiences and perceptions of the participants. Data saturation was achieved after 20 interviews were completed. There are no participants who leave the study until the research process is complete.

### Interview questions

Semistructured interviews were conducted using guidelines to generate dialogue that discussed the difficulties and feelings experienced by remote area nurses during the delivery of care during a pandemic. The question guideline was developed based on the Theory Base Rural Nursing consisting of work beliefs and health beliefs, isolation and distance, independence, lack of anonymity, outsiders/insiders, and old people/newcomers.

The main questions are as follows:

1. How was the experience of being a nurse at the IGD Puskesmas in the village?
2. How do you feel caring for this patient?
3. What lessons can you take from this incident?
4. What circumstances can you not predict?
5. What difficulties have you faced in caring for rural patients during the COVID-19 pandemic?

### Data analysis

After the verbatim transcription the interview data were analyzed using content analysis with a conventional approach. Correspondingly, data analysis begins by reading all transcripts repeatedly to understand the whole and achieve dyeing. After that, the data were read verbatim, and a text reflecting the nurse's experience was obtained and integrated into the text, which formed the unit of analysis. Later “units of meaning” were created, which were shortened. The “units of meaning” were summarized, and notes of first thoughts, impressions, and preliminary analyzes were made by the authors (open coding). Then with axial coding, the different codes are sorted into subcategories, based on how they are linked and related.

A process of discussion and reflection was developed to build agreement between authors on how to do this to organize code into subcategories. Depending on the relationship between the subcategories that appear, fewer categories are created to group and organize the subcategories into meaningful categories to form real content. Finally, the hidden meaning or latent content of this category is formulated into one theme. Raw data themes and categories were investigated accurately and revised through constant comparison methods. To report results, examples for each category are identified from the data. The following provisions are made by the author to promote credibility.

Data from interviews were reviewed after transcription and coding by the research team. In addition, the full text of the interview transcribed together with the Nvivo 12 code (words used by the patient in the interview) was available for two participants to determine the suitability of the dialogue transcript with the participant's experience. Then, three experts in the field of qualitative study were asked to examine the interviews, the codes, subcategories, categories, and themes were extracted, and if necessary synthesis and modifications were made based on the suggestions and interpretation of the data.

## Results

Semistructured interviews with 20 emergency nurse participants were obtained. Table 1 shows that 8 (40%) were males and 12 (60%) were females with an age range from 28 to 43 years (average age 36.4 years). The majority had vocational education (75%), with long experience that ranged from 5 to 15 years (average 11 years)

**Table 1.** Interviewees' Sociodemographic and Professional Characteristics

Participant number	Coding	Gender	Age (years)	Nursing educational level	Years of experience
1	P1	Female	42	Vocational	13
2	P2	Female	43	Nurse profession	12
3	P3	Female	39	Vocational	9
4	P4	Male	33	Vocational	11
5	P5	Male	30	Vocational	7
6	P6	Female	40	Nurse profession	15
7	P7	Male	33	Vocational	5
8	P8	Male	33	Vocational	8
9	P9	Female	36	Vocational	10
10	P10	Female	30	Vocational	13
11	P11	Male	42	Nurse profession	14
12	P12	Female	38	Vocational	7
13	P13	Male	33	Vocational	8
14	P14	Female	36	Vocational	10
15	P15	Female	40	Vocational	13
16	P16	Male	42	Nurse profession	15
17	P17	Female	28	Vocational	11
18	P18	Female	40	Vocational	13
19	P19	Male	42	Nurse profession	15
20	P20	Female	28	Vocational	12

In this semistructured interview, participants were asked to explain or describe their experiences as a public health center nurse who served in the emergency room during the COVID-19 pandemic. Table 2 describes the findings of three themes and seven subthemes. The three themes are compliance increases using PPE, focus to refer to, and triage at the forefront.

**Table 2.** Themes and Subthemes Derived from Analysis of Interviews

No.	Theme	Subtheme
1	Expressions of care	• Feelings through sadness • Guilt toward family • Fear of catching COVID-19
2	Compliance increased using the PPE	• Fear of catching it • New service operational standards • PPEs are limited
3	Focus to refer to	• First aid • Inadequate isolation space
4	Triage at the forefront	• Patient honesty • Triage at the forefront

PPE, Personal Protective Equipment.

### Theme 1: Expressions of care

Since the COVID-19 broke out, nurses at public health centers have been in charge of handling COVID-19 patients. Emotionally, dealing with COVID-19 patients in the countryside is not easy. Every time you enter the treatment room trying to control yourself. He does not want to look worried in front of his patient. He also felt afraid of contracting because he was always dealing with an unseen virus and patients who did not admit it during the assessment. Besides that, they also feel guilty toward their families at home, because they are people who are at high risk for infection.

#### Subtheme 1: Feelings through sadness

Feelings through sadness cannot be fooled when caring for COVID-19 patients; this arises because the virus is increasingly spreading, the patient's condition is getting worse, and the lack of public awareness. The following is the nurse's statement regarding Feelings through sadness:

we can't be sad, but actually we can't help but be sad to see conditions like this. P1, P2, P10, P11, P12, P13, P14

The declining condition of patients and the unclear vaccine, make nurses sad in what way we treat them so that they get well soon. P3, P4, P8, P9, P10

#### Subtheme 2: Guilt toward family

Guilt toward family always haunts every nurse in rural areas because they are stigmatized by their neighbors and are at the highest risk of contracting them. The following is the nurse's statement regarding guilt toward family:

we feel guilty for our parents and our family, pity them. P9, P10, P11, P12, P13, P14, P15

seeing them ostracized in society makes us sad. P1, P2, P3, P4, P5

#### Subtheme 3: Fear of catching COVID-19

Being at the forefront of handling COVID-19 is both pride and fear due to the massive spread of the virus and a lack of public awareness. The following is the nurse's statement regarding Fear of catching COVID-19:

it would be a lie if we weren't afraid. P6, P7, P8, P17, P18, P19

before leaving, always imagine that you will meet covid-19. P1, P2, P3, P9, P10, P11, P12, P13

### Theme 2: Compliance increased using The Personal Protective Equipment (PPE)

The more massive cases and rapid transmission rate of COVID-19 have made nurses in the emergency rooms even more alert. This is due to the high transmission rate of COVID-19 among health care workers as they are the front bastion of defense. Adherence to PPE is the most effective protection against COVID-19 transmission so that health workers are not infected.

#### Subtheme 4: Fear of catching it

Fear is not only felt by the general public, health care workers in the emergency room have higher level of fear, because they face patients who have not been identified as positive or negative COVID-19 virus. The following is the nurse's statement regarding the fear of contracting the COVID-19 virus:

Cases are rising, so we have to be alert. P1, P2, P10, P11, P12, P13, P14, P15

Many nurses are infected and die, so we must protect ourselves. P3, P4, P5, P6, P7, P8, P9, P10

Because the patient we are dealing with has not been detected yet. P17, P18, P19, P20

#### Subtheme 5: New service operational standards

The COVID-19 is a dangerous infectious disease that has been announced as pandemic by WHO. As a novel viral infection, it requires new action according to the new emerging evidences. The novel virus, which is different from the previous viruses, and its massive transmission are the reasons for the need for new service standards so that the infected cases can be treated effectively and health care workers are protected. The following is the nurse's statement:

This is a new and different virus. P9, P10, P11, P12, P13, P14, P15

A lot of casualties and very massive transmission. P1, P2, P3, P4, P5

Need a standard service that protects health workers. P18, P19, P20

#### Subtheme 6: PPE are limited

The risk of COVID-19 transmission in emergency departments also occurs due to the lack of provision of PPE. PPE is the main need for nurses in caring for COVID-19 patients. This resulted in nurses using other substitute PPE that under standard.

Lack of PPE assistance for us. P6, P7, P8, P9, P12, P13, P17, P18, P19

Many patients are infected. P1, P2, P3, P9, P10, P11, P12, P13

The high price of PPE. P1, P2, P3, P11, P17, P18, P19, P20

Disposable, so, can't be used for tomorrow. P4, P10, P11, P12, P17, P18, P19, P20

We use raincoats to replace protective clothing. P1, P6, P7, P8, P11

### Theme 3: Focus to refer to

The community health care centers focus on providing disease prevention services. The COVID-19 patients are referred to higher levels of health care service because community health centers cannot provide health care services according to COVID-19 patient needs due to limited facilities, services, and personnel.

#### Subtheme 7: First aid

Community health care centers focus on health promotion and prevention, but do not rule out curative activities. The care provided is the first level of health care service, and further health care actions needed for COVID-19 patient will be carried out at the referral hospital.

We can only help for first aid in patients with suspicion of Covid-19. P8, P9, P10, P11, P12, P13, P14, P15, P16, P17, P18, P19, P20

Cannot do more actions, because of the lack of facilities and infrastructure. P1, P2, P3, P4, P5, P6, P7, P8, P9, P10, P11

#### Subtheme 8: Inadequate isolation space

COVID-19 patients must get extra services, including the provision of special isolation rooms, because there is no approved treatment or vaccine for COVID-19 and the virus spreads very fast. The following is the nurse's statement:

We lack the ideal space to treat COVID-19. P12, P13, P14, P19, P20

Even the room for COVID-19 patient transit, we can't provide it yet. P1, P2, P3, P4, P5, P6, P7, P8, P9, P10, P11

### Theme 3: Triage at the forefront

Triage is a place designed to sort out patients who are suspected of having COVID-19 from other patients, so COVID-19 patients do not affect patients on other rooms. However, triage needs equipment and competent health care workers. Although selective sorting is indispensable in the triage room, it partially depends on the honesty of the patient during the assessment.

#### Subtheme 9: Patient honesty

The patient history in the triage room is very important for labeling the patient. Anamnesis becomes biased when a patient is not honest, and has an impact on treatment regimen. Moreover, lack of patient honesty has an impact on the patient himself, his family, and it is detrimental to health care workers.

Patients come lying about going anywhere. P10, P11, P12, P13, P14, P15

Patients cover their families who are at risk of COVID-19. P1, P2, P3, P4, P5, P18, P19, P20

Angry if asked more about travel history. P1, P2, P3, P4, P5, P6, P7, P8, P9

The family does not want to tell about the patient in detail. P17, P18, P19, P20

#### Subtheme 10: Selective sorting

Triage is an effective way in providing health care services. Selective sorting facilitates providing an excellent service in community health centers. Selective sorting of COVID-19 cases is needed even if the patient or family lies. The following is the statement of the nurse:

Need to know the patient or family lied while in anamnesis. P1, P2, P3, P4, P5, P6, P7, P8, P9

Comprehensive sorting to be more selective. P9, P10, P11, P12, P13, P19, P20

Triage officers must be competent to be more selective. P7, P8, P9, P10, P11, P12, P13, P14, P15

## Discussion

This study aimed to explore the experiences of emergency nurses in public health care centers during COVID-19 pandemic in Malang, East Java, in Indonesia. Few qualitative studies have been undertaken to explore the experiences of emergency nurses as a frontline health care providers during the rapid spread of COVID-19 across the globe, including Indonesia. The findings highlighted the challenges faced by emergency nurses in public health care center, which become the frontline community health care facilities accessed by patients in Indonesia.

Caring for patients with COVID-19 in rural areas is a different challenge from cities.^23^ The expression when knowing about the patient's condition makes the nurse sad and the guilt and fear of being infected causes different challenges. This is in line with this study that caregivers of chronic diseases expressed a variety of feelings and expressions from the first time they knew the diagnosis to continuing to care for at home and starting life adjustments.^24^

Since March 2020 when the first case of COVID-19 confirmed in Indonesia, around 26 Indonesian nurses died because of taking care of patients with COVID-19.^25^ As the pandemic accelerates, access to PPE for health workers becomes a key concern.^12^ This condition has improved the compliance of nurses in using PPE.^26^ There are two main reasons reported by nurses have increased their compliance in using PPE in this study: fear to be infected from the patients and PPE has become new standard operating procedures for health care workers in treating patients with COVID-19.^27^

The highest viral load of SARS-CoV-2 is in phlegm and upper respiratory tract secretions, the virus predominates spread through droplets and contact lines. Airborne precautions are recommended during aerosol-generating procedures and after this into the air. The exchange has reduced the virus considerably. The outfit comes with a plastic dress and gloves were changed between patients.^28^ According to WHO guidelines, according to PPE when providing care to COVID-19 patients are gloves, masks, protective eyewear and long-sleeved gowns with N95 respirators recommended over masks for AGP.^5,6,12,29–31^ Centers for Disease Control and Prevention recommends disposable patient isolation gown, which is used for routine patient care in health care settings, suitable for use by health care workers when treating for patients with suspected or confirmed COVID-19.^11,32^

While in isolation, patients receive many technically sophisticated nursing interventions, but restrictions on access to urban areas and the use of PPE by health care professionals have hindered the therapeutic relationship between patient and nurse. This is supported by the existing literature, which shows that the emotional impact of isolation (not only for health care professionals but also for patients and families) increases the workload for nurses and the level of stress they feel.^33^

In our study almost all nurses had concerns about the safety of their patients and themselves. This findings support previous research on the coronavirus outbreaks that show that safety is the main concern of the staff. The main strategies that help relieve nurse assistant stress due to infection are education and training. To reduce the risk of infection and the safety of yourself and occupants, nurses learn proactively preventive measures, which includes disinfection and personal hygiene. Similar findings have been reported in other studies. Problem-focused coping strategies are widely used coping strategies because they provide a sense of control through careful planning. This strategy is effective for individuals whose stress factors are under their control and can therefore manage stress factors effectively. These strategies need to be applied during nurse education and training.^7^

The Indonesian government has officially appointed more hospitals across the country as referral ones for patients infected by COVID-19 after a report on the first death of a patient. The number of the referral hospitals rise to 132 from about 100, and they are located in all the country's 34 provinces. Indonesian government has called on Indonesian hospitals to improve the referral system and treatment management of COVID-19 patients in a bid to prevent the overload of patients that were increasing lately. The government asserted that the COVID-19 referral hospitals must only treat patients who developed severe symptoms.^34^

The Indonesia Ministry of Health explained that the Community Health Center (primary health care) applied two ways to do tracing to get COVID-19 positive patients with symptoms. The first way is through the rapid test and the second through the polymerase chain reaction (PCR) method.^35^ If there is a positive result by Community Health Center, the patients are managed by two methods. First, symptomatic patients will be referred to the COVID-19 referral hospital.^18^ However, positive patients without symptoms, the public health care center will apply the protocol according to the Ministry of Health guidelines namely self-isolation.^36^

The third focus point reported by nurses in this study was about triage system in emergency unit in public health care centers. Triage system in emergency unit has become the key point in screening patients who come to seek health care during COVID-19 outbreak.^31^ According to nurses in this study, there were two main points that could enhance the effectiveness of triage implementation in emergency unit of public health care centers: patient honesty and selective selection.^37,38^

During this pandemic, honesty is the best policy. Patients who have not fully disclosed their travel history,^39^ the state of their health, or symptoms similar to those known to be related to COVID-19 have exposed medical workers to the virus, leading to bigger problems. Several doctors in Edmonton are speaking up about patients who are putting others at risk by not being honest about their potential COVID-19 symptoms or travel history.

## Conclusion

This study explored the experiences of emergency room nurses in community health care centers that may be useful for future services. This study found expressions of care, the importance of personal precaution equipment, triage, and referral system for the COVID-19 cases in the emergency rooms of the community health center. In short, this research reveals that compliance increased using PPE, focus to refer to, and triage at the forefront is the main theme identified in this study. Further investigation of the readiness of nurses in handling patients in the emergency room will be of benefit to the results of this study.

## Data Availability

The data sets used and/or analyzed during this study are available from the corresponding author on reasonable request.

## Abbreviations Used

COVID-19: coronavirus disease
PPE: Personal Protective Equipment
SARS-CoV-2: severe acute respiratory syndrome corona virus 2
